# Some Interesting and Probably Original Surgical Procedures

**Published:** 1912-09

**Authors:** A. F. Sampson

**Affiliations:** San Francisco, Cal.


### TEXAS MEDICAL JOURNAL.

Established July, 1885

F. E. DANIEL, M. D., - - - - - Editor, Publisher and Proprietor

Published Monthly.—Subscription, $1.00 a Year.

VOL. XXVIII. AUSTIN, SEPTEMBER, 1912. No. 3.

The publisher is not responsible for the views of the contributors.

### Original Articles.

For Texas Medical Journal.

### Some Interesting and Probably Original Surgical Procedures.

BY DR. A. F. SAMPSON, SAN FRANCISCO, CAL.

In my earlier days of surgical work the Whitehead operation for hemorrhoids impressed me most favorably until one of my patients upon whom I had done this operation came near dying from concealed or internal hemorrhage. This put me to considering wherein lies the defect in the Whitehead method. I saw the oversight in not giving proper consideration to ligating the hemorrhoidal vessels. I modified this operation by placing in the ligatures, so this element of danger was met. Then I mentioned to the Professor of Surgery of the University of Texas, who had been Whitehead's assistant, that I had modified and perfected Whitehead's operation for hemorrhoids. He, at first, considered me very presumptuous, etc. It was not long after our conversation that he lost a case from concealed hemorrhage following Whitehead's operation. This brought about a closer relationship between us, and he presented a paper on Whitehead's operation with an addenda from me perfecting this operation. This was read before the Medical Association of Texas and was published later in the TEXAS MEDICAL JOURNAL.

Many operations have I made with this modification for hemorrhoids without one case going bad. The correction that suggested itself to me whereby the dangers of hemorrhage should be met was using a perineal needle threaded with long catgut, after the dissection of the pile-bearing area was completed. Passing this needle thus threaded from the bottom of the dissection about onequarter of an inch from the median lateral line through to the mucosa into the bowel just superior to the internal sphincter muscle. The loop of catgut was now caught in a hemostat forceps, the needle with the rest of the ligature withdrawn. Now the needle is again passed, as above mentioned, one-quarter of an inch to the other side of the median lateral line and the catgut removed from same. The free ends of this catgut are then withdrawn from the eye of the needle and passed through the loop, the needle withdrawn and the catgut ligature thus looped is tied, thereby controlling all the important blood-vessels of the hemorrhoidal region. The same process is repeated on the opposite side, and after trimming off this dissected pile-bearing area these long ends of the catgut that we used for hemostatic purposes are now threaded on an ordinary needle and the bowel mucosa is brought down and attached to the initial incision of the muco-cutaneous surface. Henceforth the operation of attaching the bowel mucosa to the muco-cutaneous surface of the sphincter ani is continued along the Whitehead method.

I find of late by passing into the bowel a suppository of cocoa butter with proposan, a local anesthetic, there is very much discomfort saved to the patient.

The method of amputation of the cervix uteri simplifies the operation as advised by either Prior or Schroder, and I think it worthy of consideration. It simplifies, is more expeditious and has proven more satisfactory in its ultimate results. So frequently would I experience stenotic tendencies with the other methods that I improvised the following in the hope of overcoming objectionable features. Morbid interstitial changes taking place in the cervical tissues as result of extensive inflammation following a laceration, thus vitiating through strangulation its glandular functions and reflecting manifestly upon the nervous system. I have found permanent relief to be obtained only through the removal of this diseased structure.

In performing the operations that I now do, after the patient has been properly prepared, a curettage is done and tincture of iodine applied to the endometrium and the cervical canal, also to the cervix and the upper parts of the vaginal canal.

The cervix is seized by a double tenaculum in the median line, above and below. With a Mayo straight scissors carried up to the internal os a median lateral incision is made on either side of this cervix. This scissors cut takes in the entire length and thickness of the cervix. The cervical flap held apart by these double tenacula gives a good view of the cervical mucosa. A few hemostats properly applied easily controls bleeding, so that we have a clean field to proceed in our next step in the operation.

A few lines within the external os the cervical mucosa is cut through with a knife in what we estimate its underlying tissue; longitudinally an incision is made along the cervical mucosa to an equal depth along a line of the cervical canal up to the inner os. Similar incisions are made on the other side of the lower flap. The dissection of this cervical flap from the underlying diseased cervical tissue is readily accomplished. We now, at a medium point of this cervical flap, that has been dissected, pass a No. 2 ten-day chromicized catgut. The ends of this ligature are caught in a hemostat, the same process of dissecting the cervical flap is repeated on the upper half. The cervical masses we now proceed to amputate with a knife, near the cervico-vaginal junction, an incision through the vaginal surface of the cervix down and just to the diseased cervical tissue. With a scissors this diseased cervical mass that is retracted with a double tenaculum is cut out. The same process is repeated with the upper half. The next step of this operation is to pass two chromic catgut ligatures through the distal end of the muco-cervical flap to the corresponding point of the vaginal mucous lines of incision, making three stitches in each flap that serves, when tied, to bind the distal end of the muco-cervical flap to the vaginal flap. Before tying the stitches, however, all clots have been properly cleansed from the opposing surface. This method of preserving most of the cervical mucosa overcomes the obstacle of attresia to the outlet of the uterus. We now proceed to pass three deep chromic catgut stitches on either side of our median cervical stitches. All blood clots having been removed from the lateral wound, we take the first stitch with a good-sized, full curved Mayo needle on the external angle of the wound, the second about the middle of the lateral wound and the third close up to the cervical flap. These stitches are passed the full depth of the wound, generally from below upward, brought together and tied, giving the desired result. A similar process is repeated on the other side of the lateral wound, thus completing the operation. Once seen performed the advantages of this method from several standpoints can not fail to impress favorably the surgeon, and the results have proven most gratifying to my patients, no disturbances whatsoever following.

I have attended patients in parturition after the above operative procedure had been made, and wished for no better conditions to exist.

### FENESTRATED BI-VALVE URETHRAL SPECULUM.

In about 1885 in Galveston a patient consulted me; a young man with what impressed me as a condyloma about the size of a small pea forming about one and one-half inches within the urethra. To see what this growth was gave rise to the thought of dilating, without obstructing the view, with two hairpins, the blunt end passed in and so crossed that, with a head mirror to illuminate, I obtained a full and satisfactory view of the first two inches of this urethra and the full character of the growth. It was an easy matter then with the use of a small wire snare to remove said growth from the patient. This thought suggested to me a bi-valved, well fenestrated urethral speculum, for the purpose of observing the first three inches of the male urethra. Sending a diagram and description of such an instrument as I desired to Tieman & Co., instrument makers, of New York, I had two of these specula made; one I submitted to Professor Otis, specialist, for his criticism; the other I am still using. Professor Otis was very generous in his criticism. He stated the uniqueness and varied advantages of this instrument, and prevailed upon me to have a cut and description published in the *New York Medical Journal.* The special advantages I have in many years derived from the above instrument were the ease with which the anterior urethral structures could be studied, with a specially devised knife I had made the anterior stricture bands could be thus cut without unduly mutilating the urethra.

### RETROVERSION OF THE UTERUS.

The surgical methods that are done in correcting this condition attracted my attention long since. It has been my observation when a uterus has for any length of time been retroverted and congestion of this organ takes place we generally find disturbances arising in its adnexa, so that when opening the pelvic cavity so frequently find the cystic ovaries, adhesions, and tube disturbances also calling for correction. For a good many years I did modify Alexander's method of correcting uterine retroversion. Convinced that this disturbance essentially arose from the weakening and attenuation of the distal end of the round ligaments with its attachments, I would cut down over the canal of Nuck, catch up the attenuated ligament, carefully pull down the round ligament until I had secured the intra-peritoneal portion of this ligament found sufficiently strong to rely upon. This loop was brought into the external wound; traction thereupon bringing the uterus up into its proper position. The next step was to anchor this loop of round ligament through the pillars of the inguinal canal and connective tissue with combined chromocized catgut, completing the operation with a few surface ligatures. Though this method brought many satisfactory results, there were some features about it that appealed to me as not meeting the complications in long existing retroversions as they should be. I then resorted to Gilliam's method. My objection to this was, by piercing through the recti muscles and their fascia, after clearing up the uterus and its adnexa, the catching up of the round ligament properly distanced from its uterine insertion would bring through the abdominal wall the loop of ligament covered with its peritoneum, thus naturally a funnel-shaped depression would be established where the round ligament and the peritoneum passed through the parietal peritoneum of the abdominal wall. Such a condition would probably lead up to the formation of a hernia, and in this my opinion has been confirmed. The Gilliam operation gave place with me to the Mayo internal operation for uterine retroversion. There was but one point in Mayo's operation that was not altogether pleasing, viz.: In putting on his traction ligature to carry the round ligament, this incorporated the peritoneal covering as well as the round ligament, so that when the forceps that were passed through the rectal fascia muscle and into the peritoneal cavity via the internal ring following down with these forceps of round ligament that were brought out near where the ligatures grasped the ligament with its peritoneal covering. When these tissues were brought back through the internal ring there existed at this point a condition that was liable to lead up to a hernia. Since then this defect has been corrected in Mayo's method by simply incising the peritoneal covering of the round ligament where the traction ligature is to be passed. The round ligament is hooked up and freed from the adjacent tissues, and is alone brought out from the peritoneal cavity. The obviating of a funnel track leading down to the internal ring was thus accomplished with the result that has appealed to me as most ideal and meeting all the conditions a surgeon is called upon for the welfare of his patient.

IN DOING GALL BLADDER WORK there are a few observations that deserve consideration. Know that cholecystitis has its foundation as a result of infection. The mucosa of the gall bladder and the biliary ducts not only have undergone pathological changes that retain quite frequently infectious germs. These should be well considered in the treatment that follows a cholecystotomy. It is my practice two days after performing a cholocystotomy through the drainage tube that has been properly fixed in the gall bladder to begin combating the diseased condition of the mucosa of gall bladder and ducts by injecting warm saturated solutions of boracic acid with iodine added to make one per cent solution. This treatment is done daily until convinced that satisfactory conditions have been accomplished. It is an important question for us to consider wherein lies the seat of infection, that we may deal with this becomingly as surgeons and physicians. The following case may prove interesting:

A Mr. B. came to Paso Robles Springs, California, in the early part of 1906 to consult Dr. Simon Baruch, who was then visiting this place. I saw the case with Dr. Baruch. We found this man to be suffering with the symptoms of an advanced degree of jaundice; the gall bladder and biliary ducts were so disturbed that it left no doubt with us that these organs called for surgical procedure to meet such pathological conditions in his distress. The party then being told that a surgical procedure was necessary, he became so alarmed that he took the next train back to Portland, Ore. About eighteen months after this same person applied to me for relief. His condition was very much the same as when I first saw him, but he had undergone a cholecystotomy in Portland about one year previous. The patient's condition seemed desperate. I gave him the chance of an operation with the subsequent treatment. There was no gall-stone found in the gall bladder nor any stones in the ducts; however, the mucosa of the gall bladder with its thickened walls evidenced chronic cholecystitis.

The bile that was found in the gall bladder was of a vicious character. When attempting to probe out the cystic and common bile ducts, they were found almost obtruded by the results of the swelling of their mucosa. This case I kept under treatment for about six weeks, devoting special attention to washing out bladder and ducts as above mentioned. I was rewarded with a cure. This patient has not had, to my knowledge, a sick day since. He remained here so that from time to time I have seen him socially, and know that his physical condition remains correct.

There are several other cases along the lines of due consideration to the treatment of the gall bladder and the biliary ducts that should follow a cholecystotomy that could be given, but I have said enough.

